# Killer Immunoglobulin-Like Receptor Allele Determination Using Next-Generation Sequencing Technology

**DOI:** 10.3389/fimmu.2017.00547

**Published:** 2017-05-19

**Authors:** Bercelin Maniangou, Nolwenn Legrand, Mehdi Alizadeh, Ulysse Guyet, Catherine Willem, Gaëlle David, Eric Charpentier, Alexandre Walencik, Christelle Retière, Katia Gagne

**Affiliations:** ^1^Etablissement Français du Sang Pays de la Loire, Nantes, France; ^2^CRCINA, INSERM U1232 CNRS, Université d’Angers, Université de Nantes, Nantes, France; ^3^Laboratoire de Recherche et Développement, EFS Rennes, Rennes, France; ^4^L’institut du thorax, INSERM, CNRS, UNIV Nantes, Nantes, France; ^5^Laboratoire d’Histocompatibilité, EFS Nantes, Nantes, France; ^6^LabeX Transplantex, Université de Strasbourg, Strasbourg, France

**Keywords:** high-resolution killer cell immunoglobulin-like receptor typing, allele polymorphism, next-generation sequencing, International Histocompatibility Workshop DNA samples, Natural killer cells

## Abstract

The impact of natural killer (NK) cell alloreactivity on hematopoietic stem cell transplantation (HSCT) outcome is still debated due to the complexity of graft parameters, HLA class I environment, the nature of killer cell immunoglobulin-like receptor (KIR)/KIR ligand genetic combinations studied, and KIR^+^ NK cell repertoire size. KIR genes are known to be polymorphic in terms of gene content, copy number variation, and number of alleles. These allelic polymorphisms may impact both the phenotype and function of KIR^+^ NK cells. We, therefore, speculate that polymorphisms may alter donor KIR^+^ NK cell phenotype/function thus modulating post-HSCT KIR^+^ NK cell alloreactivity. To investigate KIR allele polymorphisms of all KIR genes, we developed a next-generation sequencing (NGS) technology on a MiSeq platform. To ensure the reliability and specificity of our method, genomic DNA from well-characterized cell lines were used; high-resolution KIR typing results obtained were then compared to those previously reported. Two different bioinformatic pipelines were used allowing the attribution of sequencing reads to specific KIR genes and the assignment of KIR alleles for each KIR gene. Our results demonstrated successful long-range KIR gene amplifications of all reference samples using intergenic KIR primers. The alignment of reads to the human genome reference (hg19) using BiRD pipeline or visualization of data using Profiler software demonstrated that all KIR genes were completely sequenced with a sufficient read depth (mean 317× for all loci) and a high percentage of mapping (mean 93% for all loci). Comparison of high-resolution KIR typing obtained to those published data using exome capture resulted in a reported concordance rate of 95% for centromeric and telomeric KIR genes. Overall, our results suggest that NGS can be used to investigate the broad KIR allelic polymorphism. Hence, these data improve our knowledge, not only on KIR^+^ NK cell alloreactivity in HSCT but also on the role of KIR^+^ NK cell populations in control of viral infections and diseases.

## Introduction

Hematopoietic stem cell transplantation (HSCT) provides a curative therapy for many patients with hematological malignancies (1). Donors for HSCT are currently selected based on the level of matching for HLA-A, -B, -C, -DRB1, and -DQB1 loci. Siblings, 10/10 HLA matched, remain the gold standard. However, substantial risks of morbidity and mortality caused by disease relapse (2), graft-vs-host-disease (GvHD) (3), and infection (4) are still prevalent after related, or unrelated HSCT. Natural killer (NK) cells are the first post-HSCT cells, reconstituting antiviral and antitumoral activity (5). NK cells are able to recognize the missing-self *via* killer cell immunoglobulin-like receptors (KIRs) (6). Ruggeri et al. (7) were first to report the beneficial effect of KIR ligand mismatched donor NK cell alloreactivity after T cell-depleted HLA haplo-identical HSCT resulting in less relapse, less GvHD, and better overall survival in patients with acute myeloid leukemia. The impact of KIR^+^ NK cell alloreactivity on HSCT outcome is still controversial due to the heterogeneity of graft parameters, HLA class I environment, nature of KIR/KIR ligand genetic combinations studied, and KIR^+^ NK cell repertoire size (8–12).

As HLA class I genes, KIR genes are highly polymorphic (13). In humans, 16 KIR genes have been described including eight inhibitory genes (2DL1/L2/L3/L4/L5, 3DL1/L2/L3), 6 activating genes (2DS1/S2/S3/S4/S5, 3DS1), 2 two pseudogenes (2DP1, 3DP1). These genes are located within the leukocyte receptor cluster found on chromosome 19q13.4, spanning a region of 150 kb. Within a population, the genotypic diversity of KIR genes occurs at different levels. First, the number and nature of KIR genes vary between individuals defining different KIR haplotypes. KIR haplotypes are classified into group A and group B (14). The group A haplotype is defined by a fixed set of nine KIR genes: four framework KIR genes (3DL3, 3DP1, 3DL2, and 2DL4) that form the centromeric and telomeric part of KIR locus, three inhibitory KIR (2DL1, 2DL3, and 3DL1), a pseudogene (2DP1), and a single activating KIR gene (2DS4). The group B haplotype is defined as having a variable number of KIR genes (7–14) including the four framework KIR genes and specific KIR genes (2DS2, 2DL2, 2DL5, 2DS3, and 2DS1). Second, a variable number of copies [copy number variation (CNV)] of the gene generated by recombination and replication have also been described for some KIR genes particularly those of the B haplotype (15–17). The CNV seems to influence the licensing of KIR^+^ NK cells (18). Overall, various KIR genotypes can be observed in a population. All KIR genes, and especially for inhibitory KIR, a high degree of allelic polymorphism has been described. The latest KIR Immuno Polymorphism Database (IPD–KIR) describes 753 KIR alleles. KIR allele polymorphisms need to be investigated throughout the exon and the intron regions, and regulatory regions as shown for KIR3DL1 (19). In contrast to HLA class I genes, structure and length of KIR genes vary. KIR allele polymorphisms impact both KIR^+^ NK cell phenotype and function, as we and other groups having described for KIR3DL1 (20–25) and for KIR2DL2/L3 (26). Differences in the intensity of expression (strong, weak, or null) have been described for the KIR3DL1 receptor, defining different allotypes according to the KIR3DL1/3DS1 allele combinations present in healthy individuals (21, 27). Furthermore, the nature of KIR3DL1 alleles does not only impact the KIR3DL1 cell density but also the strength of the KIR3DL1–HLA interactions which in turn can affect NK cell functions (28, 29). The recognition of KIR allotypes using anti-KIR monoclonal antibodies also varies depending on the KIR allele polymorphism (30).

Taking these points into account, it is therefore necessary to thoroughly investigate the phenotypic and functional impact of KIR allele polymorphisms. Until now, potential KIR^+^ NK cell alloreactivity in HSCT was mainly evaluated depending on the KIR/KIR ligand genetic combinations present and analyzed only at a generic level (i.e., presence or absence of KIR genes and KIR ligand). We speculate that KIR allele polymorphisms may alter donor KIR^+^ NK cell phenotype/function, and thus modulate their alloreactivity affecting HSCT outcome. However, the impact of KIR allele polymorphisms on HSCT outcome remains difficult to assess due to the lack of suitable allele typing methods for all KIR genes. Until recently, several standard methods are used to type KIR genes at allelic level. Those methods include sequence-specific oligoprobe hybridization (31–37), sequence-specific primer (SSP) typing (22), SNP assay (38), Sanger sequence-based typing (SBT) (20, 39–42), high-resolution melting (43), and also combined SSP/SBT (21, 44). KIR allelic polymorphisms have been investigated for a few functional KIR genes (KIR2DL1/2DL2/2DL3/2DS1/3DL1/3DS1). Standard methods to type KIR genes at allelic level are usually single KIR locus specific and/or target a limited polymorphism. In addition, the constant increase in the number of KIR alleles described generates more and more ambiguous KIR typing in heterozygous samples since KIR polymorphism can extend over the entire gene. Recent advances in high-throughput sequencing technology [next-generation sequencing (NGS)], especially in immunology and hematology (45), enable determination of KIR alleles and KIR gene CNV. The extent of KIR allele polymorphisms, as demonstrated by exome capture, reported 37 new KIR alleles from 15 healthy South African individuals (46). Recently, whole KIR genome sequencing by NGS was used as a control method to validate CNV genotyping in the KIR locus (17). An exome capture that focused on KIR and HLA class I loci was also recently described (47). In this study, we developed a reliable NGS method for high quality DNA samples and easily implemented for the study of KIR allele polymorphisms.

## Materials and Methods

### Samples

Thirty B-EBV cell lines from the 10th International Histocompatibility Workshop (IHW) were selected from a well-characterized panel known for their KIR gene content. KIR genotype information, including KIR allele typing of some KIR genes for all these B-EBV cell lines, was obtained either from the IPD/KIR database or from literature for specific KIR loci. Known KIR genotypes and allele typing of these 30 B-EBV cell lines are provided in the Table S1 in Supplementary Material.

### KIR Long-Range (LR) PCR and Primers

DNA genomic extractions were performed from B-EBV cell lines using a Nucleospin blood kit (Macherey-Nagel, Duren, Germany). The concentration and the purity of all DNA samples were checked on a NanoDrop 2000C spectrophotometer (ThermoFisher, Wilmington, DE, USA) by measuring the ratio of absorbance at 260 and 280 nm. In parallel, 1.5 µg of each DNA sample was loaded on an agarose gel to check the DNA integrity. For KIR LR PCR, five intergenic KIR primers already described (17) and one additional *in-house* designed primer including four forward primers (#1, 5′-gccaaataacatcctgtgcgctgctcagct-3′; #2, 5′-ctcacaacatcctgtgtgctgctaactga-3′; #4, 5′-acggctgcctgtctgcacagacagcacc-3′, #6, 5′-cacatcgtctgcaccggtcagtcgagccga-3′) and two reverse primers (#3, 5′-ttggagaggtgggcaggggtcaagtg-3′; #5, 5′-ctccatctgagggtcccctgaatgtg-3′) were used to amplify the whole KIR genome.

The KIR LR-PCR protocol was optimized using the method described by Vendelbosch et al. (17). Briefly, KIR LR-PCR was performed with 2.5 U of PrimeSTAR GXL DNA Polymerase (Ozyme, Saint-Quentin en Yvelines, France), 1× PrimeSTAR GXL buffer, 200 µM of dNTP mixture (Ozyme) and 0.2 µM final concentration of each KIR primer. The LR-PCR reaction was performed in a C1000 Touch™ Thermal Cycler (Biorad, Marnes la Coquette, France) consisted of an initial denaturation of 2 min at 94°C followed by 30 cycles of 20 s at 94°C, 12 min at 68°C and 1 cycle of final elongation of 10 min at 72°C in the final 50 µL volume. This protocol enables amplification of each KIR gene from 5′ to 3′ untranslated regions (UTR). The final KIR LR-PCR product was run on 0.7% Seakem agarose gel in TBE1X (Lonza, Verviers, Belgium) and visualized by staining with the SYBR^®^ safe (Invitrogen, Villebon sur Yvette, France) using the SimplyLoad™ Tandem DNA ladder size marker (Ozyme) to confirm the amplification and correct fragment size as well as to check for non-specific amplification.

### Library Preparation and Sequencing

Qubit dsDNA High Sensitivity Assay Kit (Life Technologies, Villebon sur Yvette, France) was used to quantify the starting DNA library in the Qubit^®^ fluorometer (Life Technologies). The library preparation was performed using the NGSgo GENDX kit (Bedia Genomics, Chavenay, France). To achieve the optimal insert size and library concentration, 250 ng of each genomic DNA was randomly fragmented according to the manufacturer’s instructions. Briefly, 8.25 µL of NGSgo master mix (prepared from 2 µL of NGSgo-LibrX Fragmentase buffer plus 3.25 µL of NGSgo-LibrX End Prep buffer plus 1.5 µL of NGSgo-LibrX Fragmentase Enzyme plus 1.5 µL NGSgo-LibrX End Prep Enzyme) (Bedia Genomics, Chavenay, France) was added to each genomic DNA in a final volume of 32.5 µL. The fragmentation, end-repair, and dA-tailing reactions were performed in a T100™ Thermal Cycler (Biorad, France) consisted of 20 min of fragmentation and end-repair at 25°C followed by 10 min of dA-tailing at 70°C. The dA-tailed DNA fragments of each sample were then subjected to adapter ligation in 9.25 µL of an NGSgo master mix containing 7.5 µL of NGSgo-LibrX Ligase mix, 0.5 µL of NGSgo-LibrX Ligation Enhancer, 0.25 µL of NGSgo-Indx adapter for Illumina, and 1 µL of nuclease free water. The adapter ligation reaction took place in a T100™ Thermal Cycler (Biorad, France) for 15 min at 20°C followed by a cooling step at 15°C. The first cleaning and size selecting of the samples after adapter ligation were performed in a 0.45× beads:DNA ratio by using the Agencourt^®^ Ampure XP (Beckman Coulter, Villepinte, France) according to the manufacturer’s instructions and eluted in 12.5 µL of 0.1× elution buffer (Lonza Rockland, USA). The size-selected, adapter-ligated DNA fragments of each DNA sample were then dual indexed with 15 µL of NGSgo reaction mix made from 12.5 µL of NGSgo-LibrX HiFi PCR mix plus 1.25 µL of NGsgo-Indx IN-5 and 1.25 µL of NGSgo-Indx IN-7 in a final volume of 25 µL followed by a PCR reaction in a T100™ Thermal Cycler (Biorad). PCR cycling was performed as follows: an initial denaturation of 30 s at 98°C followed by 10 cycles of 10 s at 98°C, 30 s at 65°C, 30 s at 72°C and 1 cycle of final elongation step of 5 min at 72°C in the final volume of 25 µL. A second DNA cleaning and size selecting was performed in a 0.6× beads: DNA ratio by using the Agencourt^®^ Ampure XP beads according to the manufacturer instructions and eluted in 16.5 µL of 0.1× elution buffer (Lonza Rockland, USA).

Quality control procedure for the library preparation included verification of fragment size before and after purification by using the QiAxcel Advanced System (QiAgen, Courtaboeuf, France). The pooled and barcoded libraries were denaturated with 0.2 M of NaOH and diluted in the pre-chilled HT1-buffer to obtain a final library concentration of 12 pM. The final denatured library was subsequently sequenced by using the MiSeq sequencer (Illumina, Biogenouest Genomics Platform Core Facility, Nantes, France; HLA Laboratory, EFS Nantes, France) with 500 cycles v2 kits, which generated 250-bp end sequence reads.

### Sequencing Data Analysis and KIR Allele Assignment

The quality of the Illumina raw data sequences obtained was monitored by using the Sequencing Analysis Viewer Illumina software. The quality of the base calling from images and sequences was determined by the quality score (Q30) which must be ≥75% for 2 × 250 bp reads. KIR reads were mapped to the human genome reference sequence hg19 (GRCh37) by using the Burrows–Wheeler Aligner Memory Efficient Mapping (BWA-MEM) tool. The binary alignment map (BAM) files containing mapped reads were then visualized on Integrative Genomics Viewer (IGV) algorithm (48).

In parallel, raw KIR sequences were aligned and visualized using the Profiler software version 1.70, initially developed by Dr. M. Alizadeh (Research Laboratory, Blood Bank, Rennes, France) for NGS-based HLA typing (49). A flowchart for data analysis using the Profiler software is provided in Figure S1 in Supplementary Material. The first step of analysis consists by merging R1 and R2 sequences to each other when at least 10 complementary bases were found between R1 and R2 of the same cluster. During this phase, for each inconsistency of base calling, the quality value was used to select the best assignment. All sequences issued from a cluster for which we could not determine complementary between R1 and R2 remained unchanged. All sequences were transformed to FASTA format at the end of this step. The second step of analysis consists of phasing each of the sequences obtained in step one by using Blast algorithm. The third step of analysis consists by merging all sequences together using Blast information. In this step, the depth for each position and the number of sequences for each allele were calculated. The first three steps are managed in a Linux environment. The last step is presentation and assignment of each construction based on database information in a friendly interface for user, all mismatches and differences to the database are extracted and presented to the user.

For KIR allele assignment, a manual bioinformatic pipeline was first used in the absence of available softwares. This consisted of exporting from IGV, all exon sequences of each KIR gene and comparing polymorphic bases with those referenced from the IPD–KIR database. Then, two different bioinformatics algorithms were used: the first one, hereafter called “BiRD,” was developed by the BiRD platform (E. Charpentier, U. Guyet, Genomics and Bioinformatics Core Facility GenoBiRD, Nantes, France) and consists of an analysis pipeline built with Snakemake on the same logic as the manual method. A flowchart for data analysis using the BiRD software is provided in Figure S2 in Supplementary Material.

#### Harvesting KIR-Specific Reads

First, raw sequences from fastq files are processed through cutadapt (v1.8.1) in order to remove Illumina adapter sequences. The cleaned reads are then mapped to hg19 (GRCh37) reference genome using BWA-MEM (v0.7.12) with the default parameters.

#### Determining Presence/Absence or KIR Genes

Absence or presence of KIR genes is evaluated using GATK DepthOfCoverage on the BAM and using a browser extensible data (BED) file describing the chromosome position of each gene (except KIR2DP1 and KIR3DP1). Coverage mean is calculated on each gene position, and a threshold of 10 is applied in order to ascertain its absence or presence. Presence/absence of KIR genes defined by NGS is concordant to the KIR genotype of the 30 IHW samples, stratified by AA vs Bx genotypes, previously validated in our laboratory by PCR-SSP multiplex method (data not shown).

#### Determining KIR Alleles

Allele-specific nucleotide positions are extracted manually using IPD–KIR alignment tool.^1^ For every gene, the Nucleotide—CDS of all alleles are aligned against the default reference allele. A python script is then used to reformat the multipage alignments in order to have one allele alignment per line. A second python script is utilized to extract all variations from the default reference allele and map the exon position number of these variations to the chromosome position. A file is created for each gene listing all the variations found for every allele. Bases at these positions are then called using SAMTools (v1.2-2) mpileup for all samples. Finally, KIR alleles are determined by calculating the percentage of nucleotide matches between the base calls and the allele variations for each KIR allele, the highest percentage giving the most confident allele.

The second algorithm used for KIR allele assignment was the Profiler software, previously described in Figure S1 in Supplementary Material, version 1.70 (49), which permits to directly assign KIR alleles at the highest level resolution (seven digits) since full intron and exon sequences were considered and also provides quality data such as mean coverage for each KIR locus. The fragment size percentage of sequences for each allele/locus was also considered as well as percentage of mapping for each KIR gene.

Overall, KIR allele assignment for each locus and for all samples corresponds to the combined KIR results obtained using manual pipelines, BiRD, and Profiler softwares. KIR alleles were assigned on the basis of the known DNA sequences identity within the IPD/KIR database.^2^ KIR alleles are named in an analogous fashion as the nomenclature used for HLA class I alleles. After the gene name, an asterisk is used as a separator before a numerical allele designation. The first three digits of the numerical designation are used to indicate alleles that differ in the sequences of their encoded proteins. The next two digits are used to distinguish alleles that only differ by synonymous (non-coding) differences within the coding sequence. The final two digits are used to distinguish alleles that only differ by substitutions in an intron, promoter, or other non-coding region of the sequence.

## Results

### LR KIR Gene Amplifications

**Figure 1:**
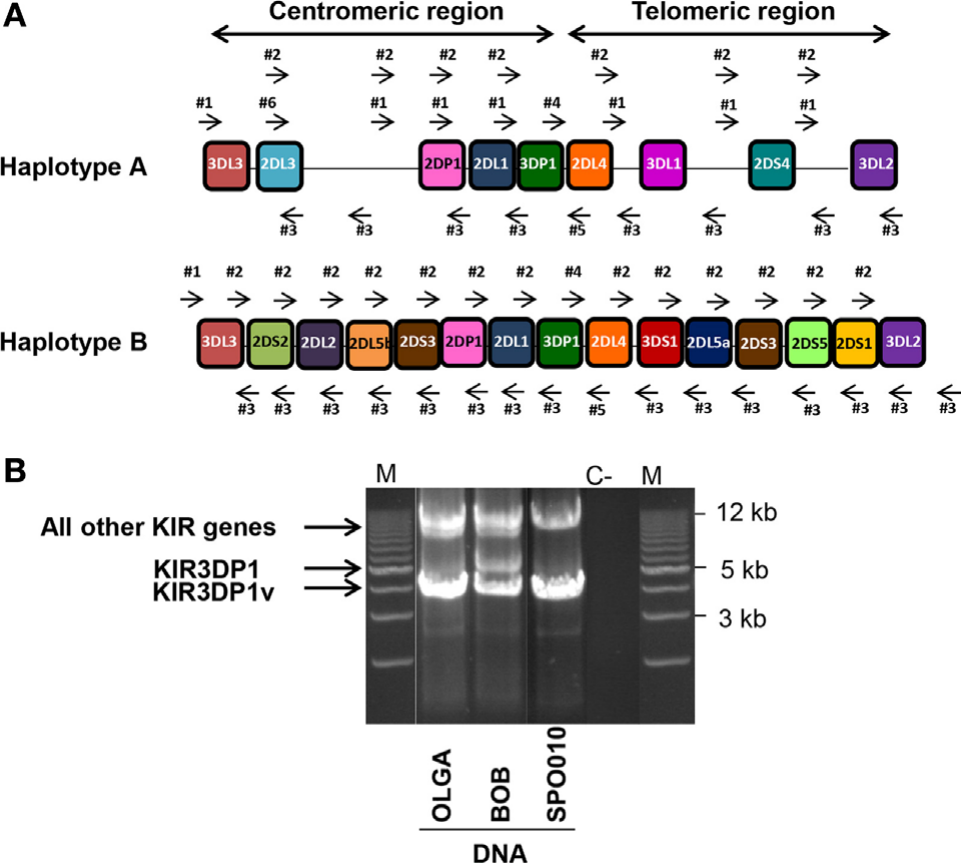
**Long-range (LR) killer cell immunoglobulin-like receptor (KIR) gene amplification**. **(A)** Six intergenic KIR primers (four forward primers: #1, #2, #4, and #6 and two reverse primers: #3, and #5) were used to perform LR PCR amplifications. These primers were able to amplify full length KIR genes in both the centromeric and telomeric regions belonging either to the A or B KIR haplotype. **(B)** Illustrative 0.7% agarose gel electrophoresis of LR PCR amplifications spanning the KIR genome of three representative International Histocompatibility Workshop (IHW) DNA samples. One IHW sample with an AA KIR genotype (i.e., SPO010) and two DNA samples with an AB KIR genotype (i.e., OLGA and BOB) were used. Amplicon lengths vary from 4 to 5 kb for the KIR3DP1 gene to 9–17 kb for all other KIR genes. Two specific bands of 4 and 5 kb corresponding to two KIR3DP1 variants were observed for BOB sample. M: Tandem ladder Lonza Seakem size marker; C^−^: H_2_O, negative control.

Thirty reference IHW samples with known KIR genotyping (Table S1 in Supplementary Material) were used to validate our NGS method for typing of each KIR gene at allelic resolution. DNA integrity, checked by loading each sample on an agarose gel, confirmed high quality for all samples (data not shown). In order to amplify all KIR genes from the 5′ UTR to the 3′ UTR, six intergenic KIR primers were chosen to allow the amplification of framework KIR genes. These intergenic primers also amplify KIR genes located either in the centromeric or telomeric region, which belong to the A and/or B specific KIR haplotype genes (Figure 1A). A robust LR amplification of KIR genes was obtained for all samples as illustrated for three representative IHW samples (Figure 1B). One specific band between 4 and 5 kb for the KIR3DP1 pseudogene and another specific band between 9 and 17 kb corresponding to a cluster of all other KIR genes were observed, irrespective of KIR AA or AB genotype (Figure 1B) as KIR genomic length varies depending on KIR genes (Table S2 in Supplementary Material). For some IHW samples such as BOB, two specific bands at 4 and 5 kb were observed for the KIR3DP1 gene corresponding to KIR3DP1*003 and KIR3DP1*001 variants, respectively, whereas only one band at 4 kb specific of KIR3DP1*003 variant was observed for OLGA and SPO010 samples (Figure 1B).

### Complete Sequencing of All KIR Genes

**Figure 2:**
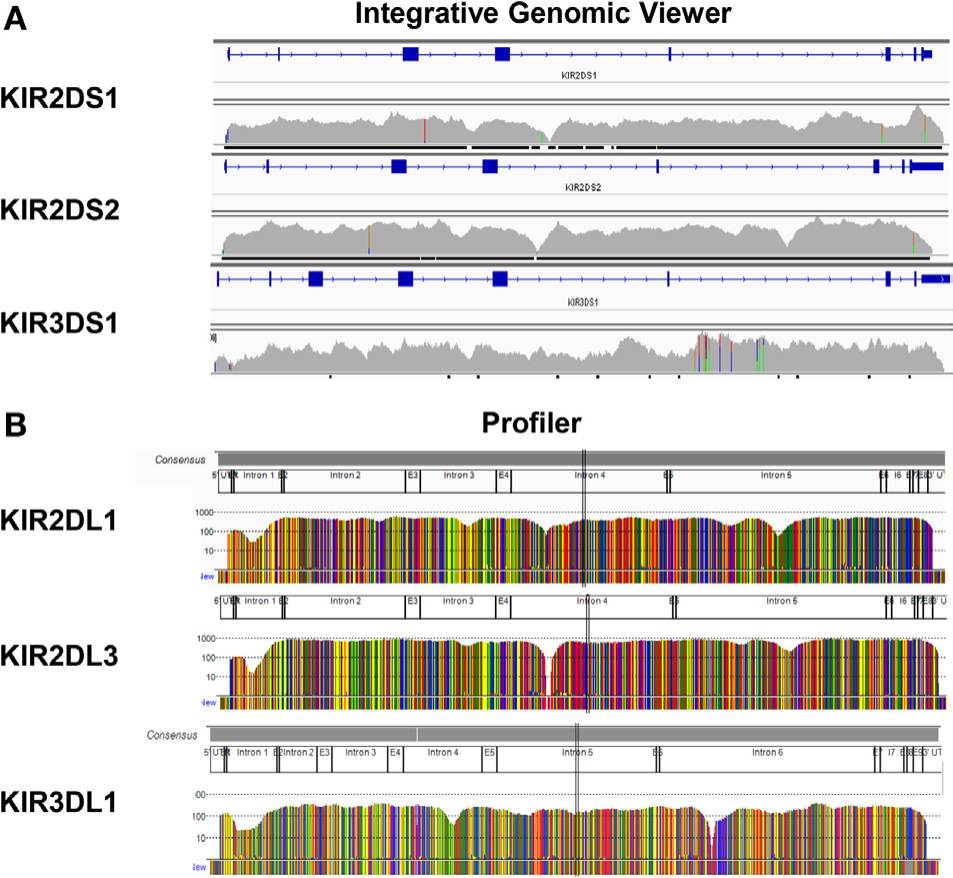
**Full sequencing of all killer cell immunoglobulin-like receptor (KIR) genes**. Reads were mapped to the human genome reference sequence hg19 using the Burrows–Wheeler Aligner Memory Efficient Mapping tool. The binary alignment map files containing mapped reads were then visualized on the Integrative Genomics Viewer as illustrated for KIR2DS1, KIR2DS2, and KIR3DS1 genes **(A)** or using Profiler software **(B)** as illustrated for KIR2DL1, KIR2DL3, and KIR3DL1 genes from one representative International Histocompatibility Workshop DNA sample.

**Figure 3:**
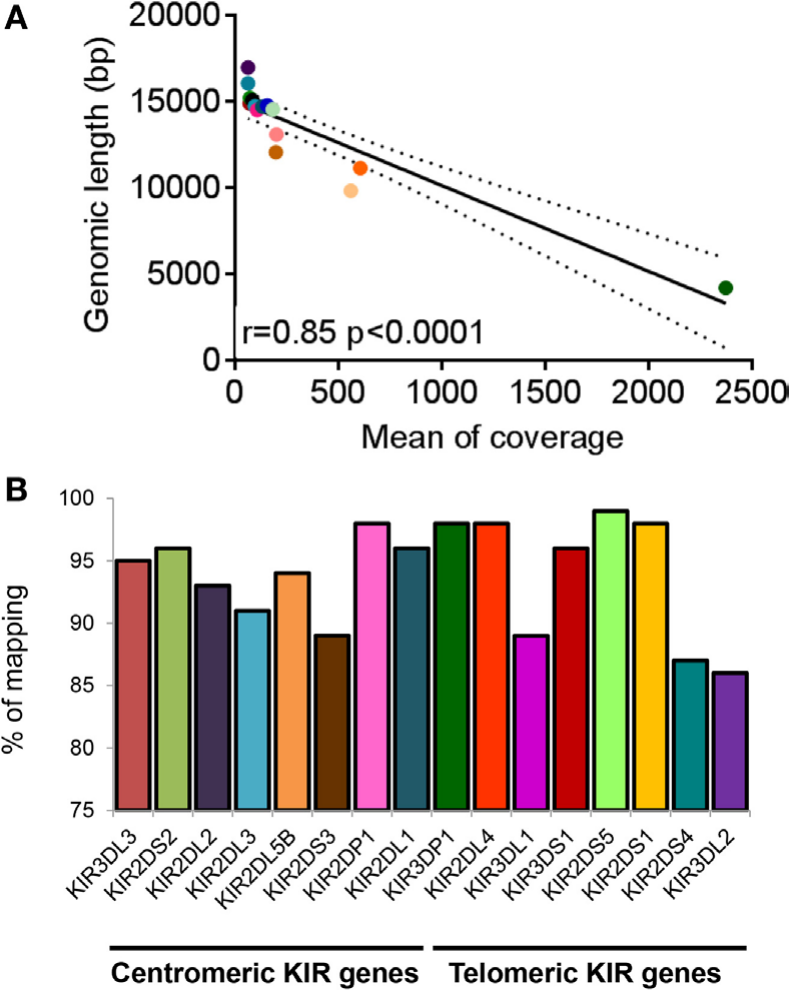
**Coverage and percentage of mapping obtained for killer cell immunoglobulin-like receptor (KIR) genes**. **(A)** Correlation graph representing mean coverage for each KIR gene and KIR genomic length. Mean coverage was estimated for each KIR gene present for all International Histocompatibility Workshop (IHW) samples using Profiler software. Statistical significance was determined using the Pearson’s rank coefficient using GraphPad Prism version 6 software (GraphPad Software, La Jolla, CA, USA). A significant *p*-value between mean coverage and genomic KIR length was observed (*p* < 0.0001). **(B)** Bars representing the percentage of mapping of each centromeric and telomeric KIR gene present for all IHW samples determined using Profiler software. KIR2DL5A locus was not included since not analyzed using Profiler.

In order to check the specificity of KIR LR-PCR obtained, amplicons were further fragmented and sequenced on paired end 2 × 250 bp from Illumina MiSeq platform. The sequencing of all amplicons yielded a total of 6.3 Gb, which was generated from a 755 ± 31 K/mm^2^ cluster density (data not shown). Approximately 88.2% of the clusters passed QC filters and on average, 82.4% of both reads passed with a Q30 > 82% (data not shown). Thus, analysis of FastQ data obtained from all IHW samples reported an excellent quality control. The entire length of KIR genes was sequenced with good coverage as illustrated for KIR2DS1, KIR2DS2, and KIR3DS1 (Figure 2A) activating genes, and for KIR2DL1, KIR2DL3, and KIR3DL1 (Figure 2B) inhibitory genes using either IGV or Profiler software, respectively. For all genes, the depth of coverage varies most at the beginning and at the end of the amplicons, but all key regions were sufficiently covered. In particular, we observed that mean coverage ranged from 62.5× (KIR2DS4) to 2,373.3× (KIR3DP1) leading to a mean coverage of 316.55× for all KIR genes except for KIR2DL5A genes since not analyzed using Profiler (Table S3 in Supplementary Material). A significant correlation was observed between mean coverage and genomic KIR length (*r* = 0.85, *p* < 0.0001) as illustrated Figure 3A. Indeed, the lower the genomic length, the higher the mean coverage is as illustrated for the KIR3DP1 gene. The mean percentage of mapping, established by the coverage of amplicon, ranged from 86.2% (KIR3DL2) to 98.2% (KIR2DP1 and KIR3DP1) (Figure 3B; Table S3 in Supplementary Material) suggesting that sufficient read depth was obtained for determination of all KIR genes. However, KIR2DL5A reads could have been mapped only using BWA-MEM software and BiRD algorithm. Overall, these results demonstrate the efficiency of our NGS-KIR allele typing approach to capture the full KIR genomic locus and the uniformity of coverage for each KIR locus confers assurance for KIR allele assignment.

### Specificity of NGS-Based KIR Allele Typing

Due to the high degree of KIR polymorphisms and the fact that NGS technology generates a lot of sequencing reads, three different algorithms were evaluated to increase the reliability of KIR allele assignment as reported for NGS-based HLA typing (50). KIR allele assignment was first done manually and then confirmed using both BiRD pipeline and Profiler software. Overall, resulting KIR allele assignments of the 30 reference IHW samples were feasible for all loci and for the majority of samples without remaining ambiguities (Table 1).

**Table 1:** **Next-generation sequencing-based killer cell immunoglobulin-like receptor (KIR) allele typings of 30 reference B-EBV cell lines from the 10th International Histocompatibility Workshop**.

ID	Centromeric KIR genes									Telomeric KIR genes								
	3DL3	2DS2	2DL2	2DL3	2DL5B	2DS3	2DP1	2DL1	3DP1	2DL4	3DL1	3DS1	2DL5A	2DS3	2DS5	2DS1	2DS4	3DL2
AMAI	*013			+			*00301	+	*003	*0080101	*001						*00301	*00101
	*041						*004		*006	*0080102						

AMALA	*00402	*00101	*00301	*001			*00201	*00302	*007	*00102	*01502		*001		*00201	*00201	*001	*0020105
	*00802								*00901	*00501		*01301						*0070102

BOB	*00101	*00101	*00301	*00201			*00301	*00302	*002	*001	*002	*01301	*00101		*00201	*00201	*001	*0020101
	*01303								*00302	*005								*0070102

BRIP	*00801	*00104	*00301	+		*00103	*0010201	*00302	+	*0010305	*008	*01301	*00103	*00103		*002	*003	*0070102
		*004				*00201	*0020101			*00501			*00501	*00201				*0070103

CALOG	*00207			+			*00201	*00302	*00302	*008	*001						*00301	*00101

ERO	*01001	*010	*004	*00601	*00301

COX	*00102			*00201			*00301	*00201	*005	*00501*	*005010	*055	*00101		*00201	*00201	*010	*00103
	*00103			*007					*006	*011								*007

DEU	*00101	*00101	*001	*00201			*00301	*00201	*001	*00801	*00101						*003	*01001
	*01402								*006	*011	*00501						*010	*01101

DKB	*00101			+			*00301	*00201	*00302	*0010201	*002						*00101	*0020101
	*006								*006	*00103	*02001						*00902

HO301	*014	*00101	*00101		*010	*00103	*00102	*004	*003010	*00102	*002			*00103			*001	*00201
		*002	*00301			*00201		*010	*004					*00201				

HID	*01402			*00101			*00201	*00302	*00302	*00102	*01502						*00101	*00201
	*018						*010											

HOM-2	*00101			+			*00201	*00302	*00302	*00801	*001						*00301	*0010102
	*0090101						*005		*006	*00802	*004						*00601	*00501

HOR	*001			+			*00301	*00201	+	*00501		*01301	*00101		*002	*00201		*007
	*048																	*021

JHAF	*00901			*00101			*002	*00302	*00302	*011	*00501						*010	*001
	*026																	*01001

JVM	*007	*00101	*00301	+			*005	*00302	*001	*00103	*00101						*003010	*00101
	*00801								*00302	*00801	*008							*009

KAS011	*00901			+			*002	*00201	*00302	*00103	*008	*01301	*00101		*00201	*00201	*00301	*01001
	*01302						*00301	*00302	*006	*005								*019

KAS116	*013			+			*002	*00302	+	*011	*00501						*010	*0103
	*01501																	*010

LBUF	*00301	+	+	*001			*002	+	*00302	*00102	+						+	+
	*0090101								*0090102	*011								

LUY	*001			*00101			*00201	*00302	*00302	*00801	*00401						*00601	*001
	*02701			*00501			*00301			*011	*00501						*010	*00501

MOU	*00207			*001			*00201	*00302	*00302	*00801	*00101						*00301	*010
	*00801						*005				*00401						*00601	*01101

OLGA	*00201			*00101			*00201	*00302	*00302	*005	*001	*01301	*00103		*002	*002	*010	*00701
	*00902						*006			*011	*00501							

PE117	*00101			*00101			*00201	*00201	*00901	*00501	*00401	*01301	*001		*00201	*00201	*00601	*00701
	*01002			*00201			*00301	*00302		*00802								*018

PF04015	*01402	*00101	*00101						*001	*011	*00501						*010	*00103
			*003															

RSH	*0040202	*00101	+	+	*004		*00201	*00302	*00304	*0010307	*00501				*006		+	+
	*00901						*009	*01201	*008	*011	*017							

SAVC	*00801			*00101			*008	*00302	*00302	*00102	*00401						*006010	2*00202
	*00202						*00201			*00802	*01502							*00301

SPO010	*00206			+			*00201	*00302	+	*011	*0050101						*010	*001

T7526	*0090101			*00101			*00201	*00302	*00302	*00501	*01502	*013	*00101		*00201	*002	*001	*0020105
										*00102								*0070102

VAVY	*002			*00101			*002	*002	*00302	*011	*00501						*010	*0010302
	*017			*00201			*003	*00302	*006									

WT51	*00103	*00101	+	+	*00201	+	*001	+	+	*00501		*01301	*00101	+	*002	*002		+
	*036						*004						*00501					

WDV	*00301	*00101	*003	+		*002	*002	*00302	*00302	*00501		*01301	*00501	*002		*00201		*0070103
	*0090101							*00901										

YAR	*00102			+			*002	+	*00302	*0010201	+						+	+
							*003		*006	*011								

*Results are presented according to the centromeric or telomeric localization of KIR genes on human genome. KIR alleles were named according to the last nomenclature available on the IPD/KIR database (http://www.ebi.ac.uk/ipd/kir/). + indicates the presence of a specific KIR gene. ID, sample identification*.

**Figure 4:**
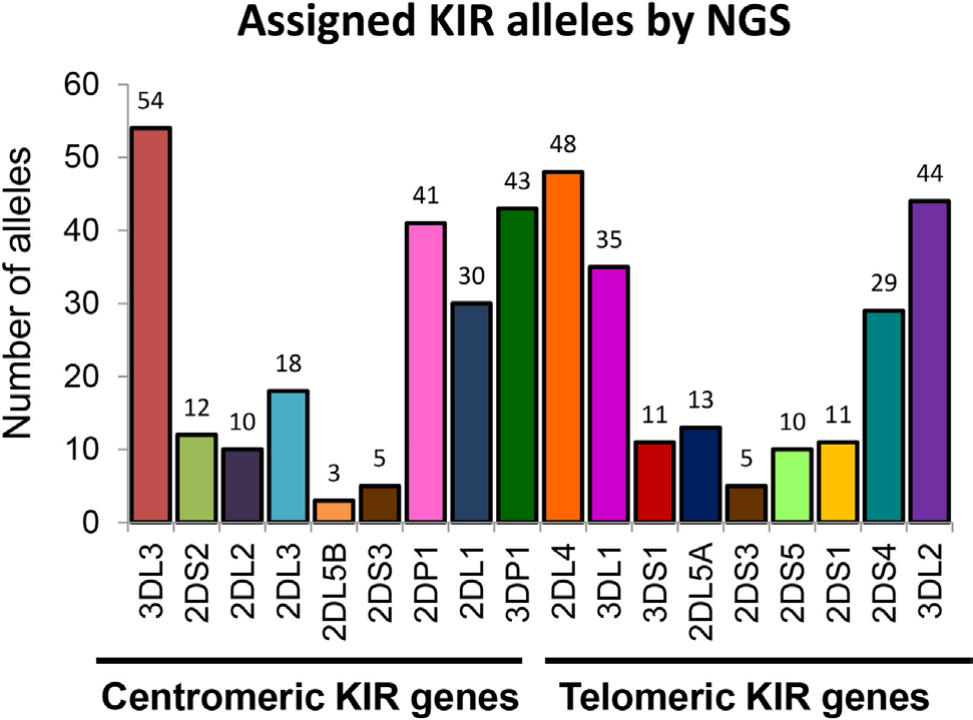
**Number of killer cell immunoglobulin-like receptor (KIR) allele assigned by next-generation sequencing (NGS)**. Number of KIR alleles assigned for each centromeric and telomeric KIR gene of 30 International Histocompatibility Workshop samples obtained by our NGS method. KIR allele assignment for each locus and for all samples corresponds to the combined KIR allelic results obtained using manual pipelines, BiRD, and Profiler software.

We further evaluated the strength of our NGS-based method for KIR allele assignment. For all IHW reference samples tested (*N* = 30), the number of KIR alleles previously known in the IPD/KIR database and those obtained by our NGS-KIR based typing approach was compared for each KIR locus. As an example, from the 30 IHW samples tested, only 5 KIR3DL3 alleles out of 60 expected alleles for this framework gene were previously known in the IPD/KIR database (Table S1 in Supplementary Material), 54 KIR3DL3 alleles from 24 heterozygous and 6 homozygous samples (Table 1) were assigned by our NGS-based KIR allele typing approach (Figure 4). Our NGS-based KIR allele typing approach permits identification of additional framework KIR alleles, e.g., KIR3DP1 (*n* = 43), KIR2DL4 (*n* = 48), and KIR3DL2 (*n* = 44) (Table 1; Figure 4). NGS-based KIR typing method also allows the identification of polymorphisms of well-functionally characterized KIR by increasing the number of assigned KIR alleles of the 30 IHW samples available in the IPD/KIR database (Table S1 in Supplementary Material), e.g., KIR2DL1 (*n* = 30), KIR2DL2 (*n* = 10), KIR2DS1 (*n* = 11), KIR2DS2 (*n* = 12), KIR3DL1 (*n* = 35), and KIR3DS1 (*n* = 11) (Table 1; Figure 4). The number of activating KIR2DS1, KIR2DS2, and KIR3DS1 assigned alleles by NGS remained low because only IHW samples with the corresponding activating KIR gene were included in this analysis. Overall, a higher number of KIR alleles were identified from these 30 IHW samples by our shotgun NGS methodology compared to those previously characterized by other less sensitive methods, as referred to in the IPD/KIR database (*N* = 422 vs *N* = 233, respectively).

**Figure 5:**
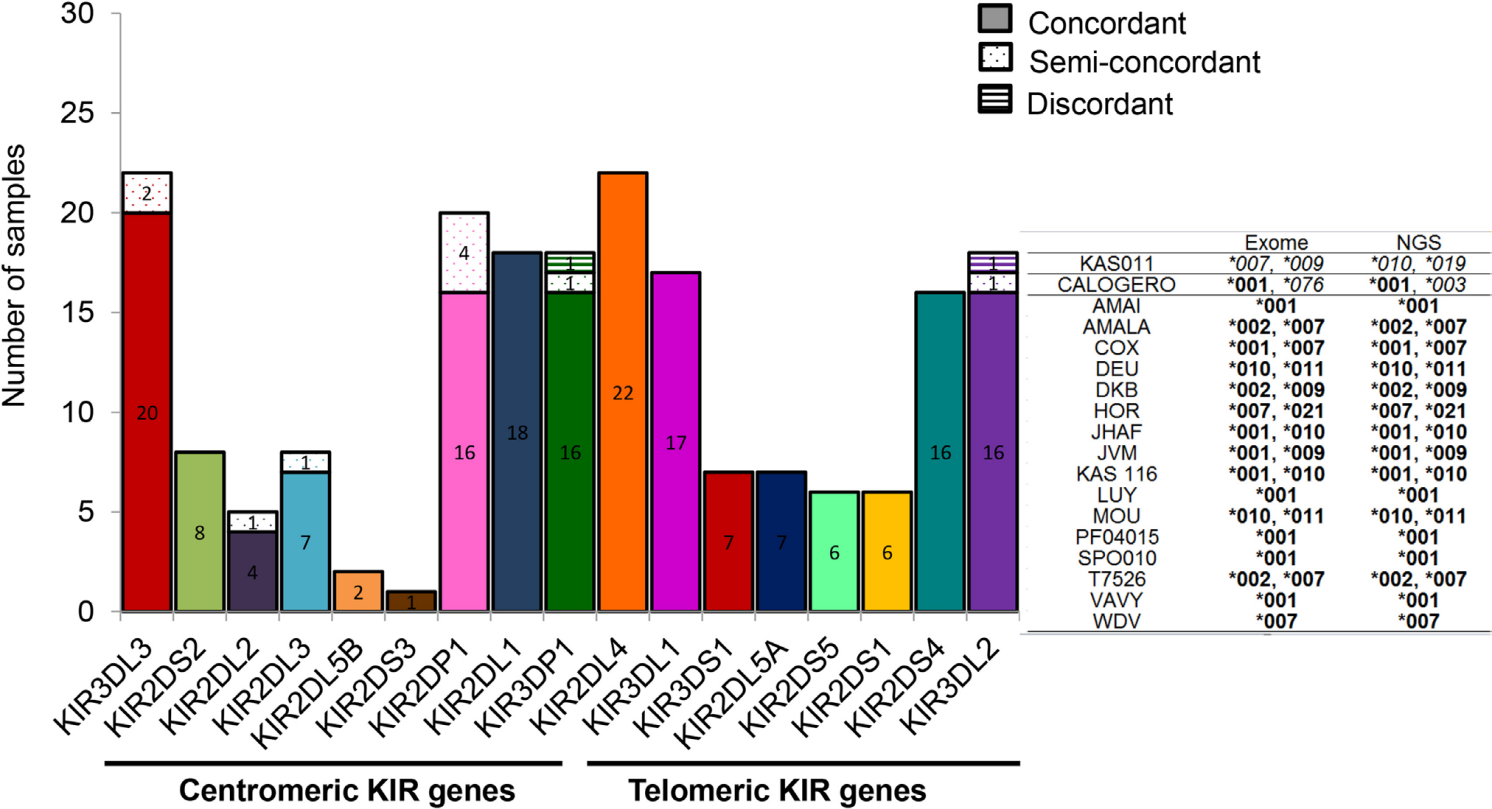
**Specificity of next-generation sequencing (NGS)-based killer cell immunoglobulin-like receptor (KIR) allele typing**. Bars representing the numbers of KIR allele typing obtained by our NGS-based method from 22 International Histocompatibility Workshop (IHW) samples compared with those assigned by exome analysis (47). In this case, KIR allele typing for each locus and for all samples corresponds to the combined KIR allelic results obtained using manual pipelines, BiRD, and Profiler softwares. Each bar represents one specific centromeric or telomeric KIR gene. Concordant (one KIR allele matched for homozygous typing or two KIR alleles matched for heterozygous typing), semi-discordant (one KIR allele mismatched for heterozygous typing), and discordant KIR allele typing (one KIR allele mismatched for homozygous typing or two KIR alleles mismatched for heterozygous typing) were highlighted by a specific color code. Representative KIR3DL2 typing of IHW samples obtained by exome analysis compared to those assigned by NGS method is provided in the right of the graphs. Concordances are highlighted in bold and discordances in italics.

The knowledge of KIR allele typing of IHW samples, recently updated thanks to an exome capture (47), permits to evaluate the concordance of our NGS-based KIR allele results (Table 1) with those of Norman et al. since 22 IHW samples were commonly used in both methods (Table S1 in Supplementary Material). In this case, a large number of allelic KIR typing for all loci was compared ensuring the reliability of our NGS-based KIR allele typing method. For each KIR locus and for the 22 IHW concerned samples, KIR allele typing results were divided into: concordant (one KIR allele matched for homozygous samples or two KIR alleles matched for heterozygous samples), semi-concordant (one KIR allele matched and one KIR allele mismatched), and discordant (one KIR allele mismatched for homozygous sample or two KIR alleles mismatched for heterozygous sample). For each KIR allele, only the first three digits were taken into account for the assessment of concordance. Complete concordance (100%) of KIR allele typing was demonstrated in 11 KIR genes. The concordant genes were KIR2DS2 (8 samples out of 8), KIR2DL5B (2 out of 2), KIR2DS3 (1 out of 1), KIR2DL1 (18 out of 18), KIR2DL4 (22 out of 22), KIR3DL1 (17 out of 17), KIR3DS1 (7 out of 7), KIR2DL5A (7 out of 7), KIR2DS5 (6 out of 6), KIR2DS1 (6 out of 6), and KIR2DS4 (16 out of 16) (Figure 5). Concordant results were observed, but at a lesser frequency for KIR3DL3 (20 out of 22, i.e., 91%), KIR2DL2 (4 out of 5, i.e., 80%), KIR2DL3 (7 out of 8, i.e., 88%), KIR2DP1 (16 out of 20, i.e., 80%), KIR3DP1 (16 out of 18, i.e., 89%), and KIR3DL2 (16 out of 18, i.e., 89%) (Figure 5).

Ten semi-discordant KIR allele results and two discordant KIR allele results between our NGS-based method and exome data were identified (Table 2). Except for the pseudogene KIR2DP1, with four IHW samples, these discrepancies were limited to 1 or 2 out of 22 IHW samples per locus (Table 2). KIR allele determinations using manual, BiRD algorithm, and different versions (the latest one Rev 2.0.188) of Profiler software were carefully reviewed. Only IHW samples sequenced on different runs and with the same KIR allelic results were reported (data not shown). These potential discrepancies (5%), possibly linked to the design and implementation of each algorithm, need to be further validated by another typing method such as SSP or sequencing.

**Table 2:** **Discordant killer cell immunoglobulin-like receptor (KIR) typing of International Histocompatibility Workshop samples observed between typing obtained by exome capture^a^ (47) and those obtained by next generation sequencing (NGS) in this study**.^b^

ID	Centromeric KIR genes						Telomeric KIR genes					
	3DL3		2DL2		2DL3		2DP1		3DP1		3DL2	
	Exome KIR typing^a^	NGS typing^b^	Exome KIR typing^a^	NGS typing^b^	Exome KIR typing^a^	NGS typing^b^	Exome KIR typing^a^	NGS typing^b^	Exome KIR typing^a^	NGS typing^b^	Exome KIR typing^a^	NGS typing^b^
AMAI							*003, *013	*00301, *004				

CALOGERO	*00207, *017	*00207, *01001							*002, *00302	*00302, *010	*00101, *076	*00101, *00301

COX					*002	*00201, *007						

DKB									*015	*00302, *006		

KAS011							*002	*002, *00301			*00701, *00902	*01001, *019

LUY							*002, *016	*00201, *00301				

PF04015			*003	*00101, *003								

WT51							*004, *018	*004, *001				

YAR	*00102, *044	*00102										

*Discrepancies are shown in italics for each KIR locus concerned. Allelic typing in bold represent concordant alleles. KIR alleles were named according to the last KIR nomenclature. ID, sample identification*.

Overall, our NGS-based method and exome data showed a rate of concordance of 95% for all loci, established for all KIR genes on 22 IHW samples, suggesting a reliable method.

## Discussion

In this study, we developed an NGS-based KIR allele typing approach to characterize the sequence of all polymorphic KIR genes. Our method of typing all KIR genes at high resolution provides an alternative, easily implemented method practice, to study the KIR allele polymorphisms. It may be a cheaper method than exome capture (47). This tool is currently adapted to the KIR gene large-scale analysis. Using our approach, the majority of KIR alleles previously uncharacterized by standard methods were clearly identified from genomic DNA of 30 B-EBV cell lines from the 10th IHW. High quality DNA samples, high fidelity of enzyme polymerase, and a reliable library preparation were needed since evaluation of different Taq polymerase enzymes and library preparation kits gave conflicting results (data not shown).

Our study evaluated the performance of different algorithms for KIR allele assignment. Reliability of the manual, BiRD pipeline, and Profiler software was tested since neither algorithm alone was able to provide 100% accuracy for all KIR loci. Our results showed that the Profiler software was reliable to assign KIR alleles through the full length of each KIR gene, excluding KIRDL5A variants. In this case, KIR2DL5A and KIR2DL5B sequences were too closed, and Profiler software failed to accurately analyze both sequences. Since all coding, non-coding, and regulatory regions were explored, one could expect that a lot of new KIR alleles will soon be described. Analysis with Profiler consists of two distinct parts. The first part is performed in three steps in a Linux environment: the first step corresponds to the merging of each R1 and R2 issued from the same cluster to each other, each time that a complementarity of at least 10 bases is found, with correction or base calling inconsistencies using a quality value for each nucleotide. There are two interests in this step: longer sequences and lower sequences number were analyzed. The second step corresponds to the phasing of each sequence based on KIR databases using Blast algorithm. Third, the data file from Blast was used to merge all sequences together to construct each allele. In this step, calculation for depth of each position and the number of sequences used for each allele are determined. The second part is done on a Windows environment. A friendly interface presents graphics of all sequences for all studied loci. Assignment of all sequences is done using a database, highlighting all mismatches compared to reference and also differences between KIR alleles selected. Each allele is scored for quality control as per the European Federation for Immunogenetics guideline.

Killer cell immunoglobulin-like receptor alleles of all genes including KIR2DL5A, but excluding the pseudogenes KIR2DP1 and KIR3DP1, were assigned using BiRD algorithm. However, many allelic ambiguities remained when this pipeline was used alone (data not shown). It is likely that this is due to the fact that only coding regions (CDS) were taken into account for allele variation comparison. Analysis of all exon/intron polymorphisms, CNV detection, summary statistics of call accuracy for KIR gene content (presence/absence) and for KIR allele identification needs to be completed. Furthermore, the two pseudogenes KIR2DP1 and KIR3DP1 could be manually added to the BED file describing the gene positions on the genome in order to include them in the analysis pipeline.

Due to the time-consuming nature of manual KIR allele assignment, two different algorithms are needed to ensure the reliability of NGS-based typing methods for the identification of KIR allele polymorphisms.

Until now, KIR genetic population studies have often been restricted to the identification of KIR gene content, or of A and/or B KIR haplotypes (51, 52). Determination of KIR alleles in healthy individuals of a given population may provide a better definition of KIR haplotypes (52) and KIR gene linkage disequilibrium (53) and will considerably increase the IPD/KIR database.

The implementation of our suitable NGS.KIR method will enable analysis of all allelic polymorphism within KIR genes extending to all coding, non-coding, and regulatory regions. A link between KIR allelic polymorphism and the expression level and/or function of the corresponding KIR^+^ NK cells is necessary for all KIR genes as previously established for the expression level of HLA-A and HLA-Cw molecules (54–56). We speculate that KIR allelic polymorphisms may affect not only the distribution and function of these gene products but also the licensing of NK subpopulations as described for HLA class I molecules (57, 58). Deep analysis of KIR^+^ NK cell phenotype and function depending on KIR and HLA class I alleles present is needed to assess the diversity of KIR^+^ NK cell repertoire (21, 59), as well as the specificity of anti-KIR antibodies (30, 60). Overall, the analysis of KIR allelic polymorphisms combined with the autologous HLA class I environment will enable better evaluation of KIR^+^ NK cell functional subpopulations (61). This functional KIR^+^ NK cell repertoire will be better defined by taking into account the nature of KIR alleles present in addition to the autologous HLA class I environment.

Investigation of KIR allelic polymorphism may be of an immunological interest in the context of viral infections such as those related to CMV (62), HIV (63), HCV (64), and of human reproduction (65). In the context of HSCT, inclusion of KIR allele typing in addition to HLA typing may provide a better evaluation of HSC donor’s KIR^+^ NK cell repertoire (21, 59, 60, 66, 67). An identification of those with the best antileukemic potential will provide a potential tool to determine an early posttransplant hematopoietic chimerism when donor and recipient have identical KIR genotypes (68) as well as the impact of KIR^+^ NK cell alloreactivity on HSCT outcome (69–73). The functional relevance of typing both KIR and HLA genes at high resolution may help determine their combined effects on outcome of HSCT.

## Author Contributions

BM performed KIR allele typing by next-generation sequencing, KIR allele assignment, interpretation of data, and wrote the manuscript. NL performed DNA extractions from EBV-B cell lines, KIR genotyping and KIR allele typing by next-generation sequencing and commented on the manuscript. MA upgraded Profiler software integrating a KIR module, analyzed data, and commented on the manuscript. UG developed a pipeline for KIR allele assignment, analyzed data, and commented on the manuscript. CW and GD performed DNA extractions from EBV-B cell lines and commented on the manuscript. EC supervised the development of a pipeline for KIR allele assignment, provided a bioinformatic help on KIR read mapping, and commented on the manuscript. AW provided advices on the library construction setting and commented on the manuscript. CR designed the study, analyzed and interpreted data, commented on the manuscript, and contributed to writing the manuscript. KG designed the study, analyzed data, and wrote the paper. All the authors have approved the manuscript for publication.

## Conflict of Interest Statement

The authors declare that the research was conducted in the absence of any commercial or financial relationships that could be construed as a potential conflict of interest.

## Footnotes

^1^https://www.ebi.ac.uk/ipd/kir/align.html.

^2^http://www.ebi.ac.uk/ipd/kir/alleles.html
