# Supplementary material for: Killer Immunoglobulin-Like Receptor Allele Determination Using Next-Generation Sequencing Technology
Source: Front Immunol. 2017 May 19;8:547. doi: 10.3389/fimmu.2017.00547 (PMC5437120; doi:10.3389/fimmu.2017.00547)
Supplement: Supplementary file 1 [file image_1.pdf]

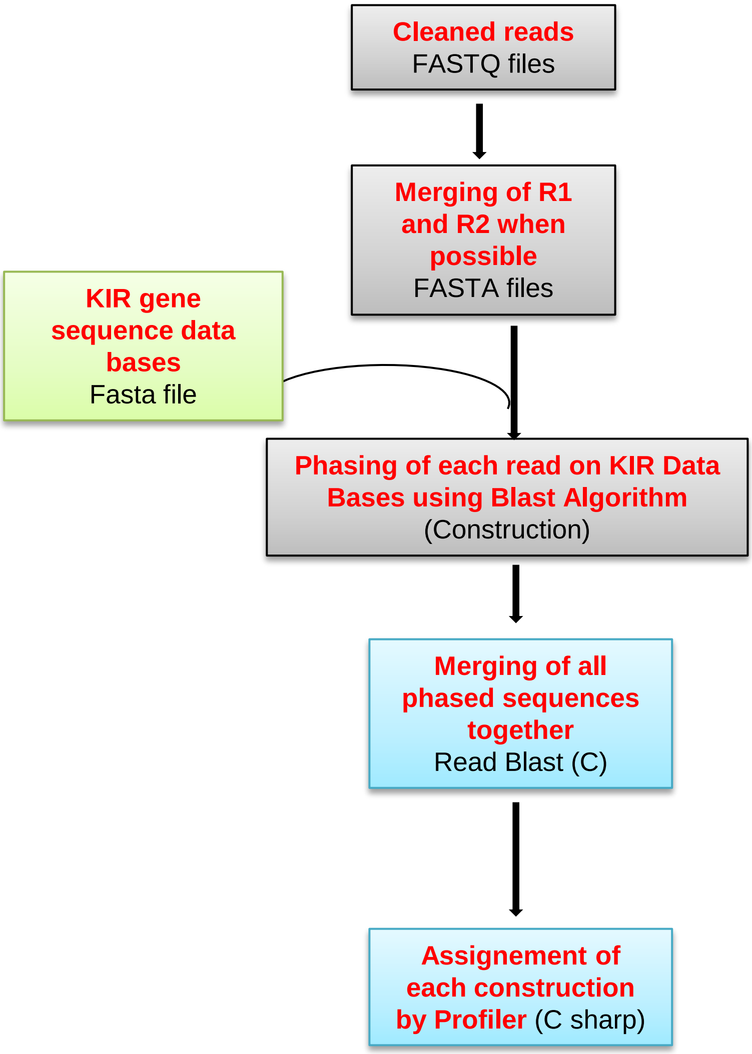

Supplemental Figure 1: Profiler pipeline description. Grey boxes represent files resulting from the main steps of the pipeline. Green boxes correspond to public data files. Blue boxes correspond to algorithm developped by EFS written in C language and C sharp.
