# Supplementary material for: Killer Immunoglobulin-Like Receptor Allele Determination Using Next-Generation Sequencing Technology
Source: Front Immunol. 2017 May 19;8:547. doi: 10.3389/fimmu.2017.00547 (PMC5437120; doi:10.3389/fimmu.2017.00547)
Supplement: Supplementary file 2 [file image_2.pdf]

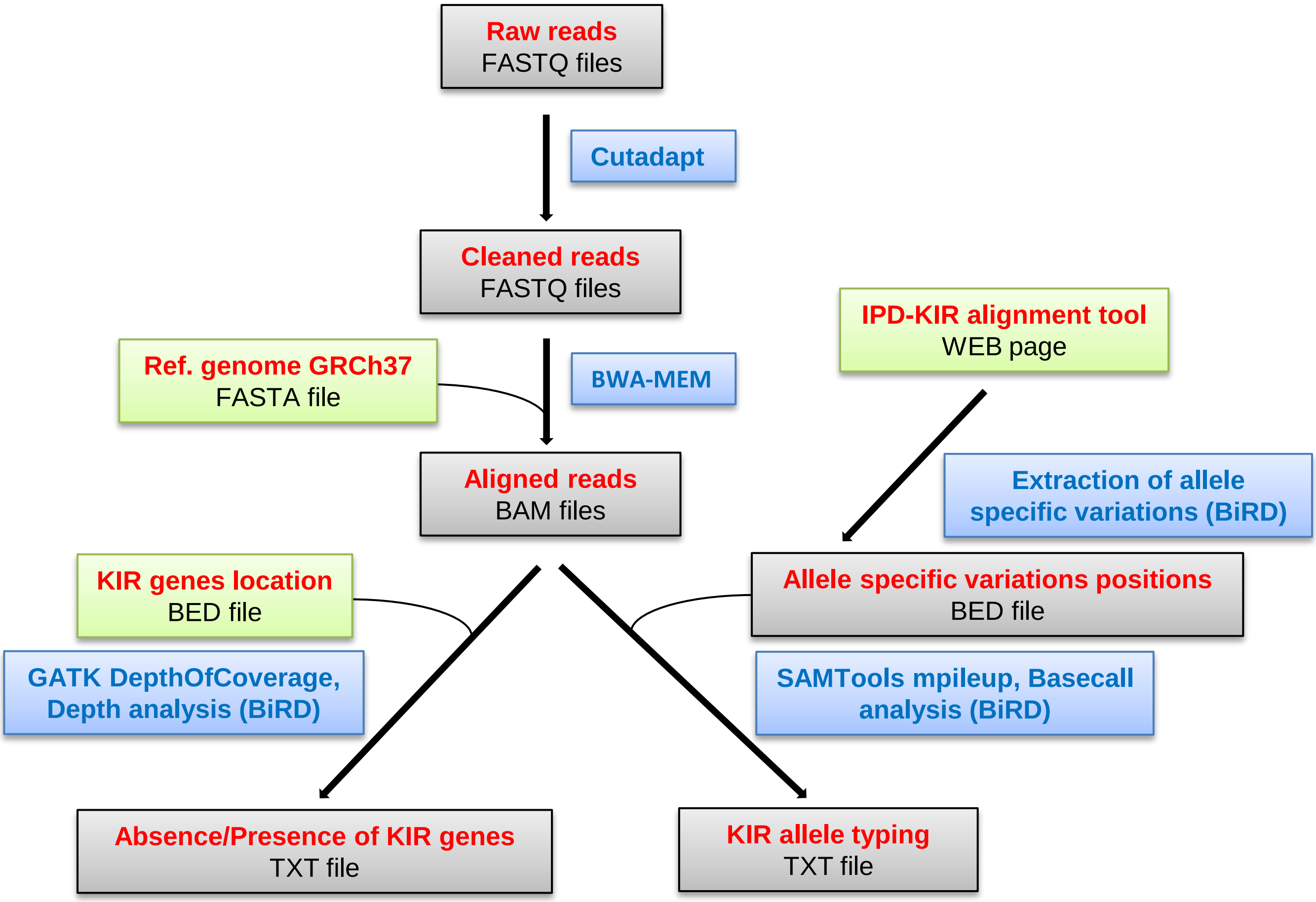

Supplemental Figure 2: BiRD pipeline description. Grey boxes represent files resulting from the main steps of the pipeline. Blue boxes correspond to publicly available bioinformatic NGS tools, and scripts developed on BiRD platform; Green boxes correspond to public data files. The pipeline was written in Snakemake, the workflow management system which can benefit from parallelization on high performance computing architectures
