# Supplementary material for: Killer Immunoglobulin-Like Receptor Allele Determination Using Next-Generation Sequencing Technology
Source: Front Immunol. 2017 May 19;8:547. doi: 10.3389/fimmu.2017.00547 (PMC5437120; doi:10.3389/fimmu.2017.00547)
Supplement: Supplementary file 3 [file table_1.pdf]

**Centromeric KIR genes**

| | 3DL3 | | 2DS2 | | 2DL2 | | 2DL3 | | 2DL5B | | 2DS3 | | 2DP1 | | 2DL1 | | 3DP1 | |
|---|---|---|---|---|---|---|---|---|---|---|---|---|---|---|---|---|---|---|
| ID | IPD[1] | *exome* [2] | IPD[1] | *exome* [2] | IPD[1] | *exome* [2] | IPD[1] | *exome* [2] | IPD[1] | *exome* [2] | IPD[1] | *exome* [2] | IPD[1] | *exome* [2] | IPD[1] | *exome* [2] | IPD[1] | *exome* [2] |
| AMAI | + | *01309, *041 | - | - | - | - | *002, *005 | *002, *005 | - | - | - | - | + | *003, *013 | *002, *001 | *001, *01202 | *003 | *00302, *006 |
| AMALA | + | *00402, *00802 | + | *001 | *003 | *003 | *001 | *001 | - | - | - | | + | *002 | *003 | *00302 | *001, *003 | *007, *009 |
| BOB | + | | + | | + | | + | | - | | - | | + | | + | | *001, *003 | |
| BRIP | + | | + | | + | | + | | B | | + | | + | | + | | *003 | |
| CALOGERO | + | *00207, *017 | - | - | - | - | + | *001 | - | - | - | - | + | *002 | + | *00302 | *003 | *002, *00302 |
| COX | *00102 | *00103, *00102 | - | - | - | - | *002 | *002 | - | - | - | - | *003 | *003 | *002 | *002 | *005, *006 | *005, *006 |
| DEU | + | *00101, *01402 | + | *001 | *001 | *001 | *002 | *002 | - | | - | - | + | *003 | *002 | *002 | *001, *003 | *001, *006 |
| DKB | + | *00101, *00602 | - | - | - | - | + | *002 | - | | - | - | + | *003 | + | *002 | *003 | *015 |
| HO301 | + | | + | | + | | - | | B | | + | | + | | + | | *001, *003 | |
| HID | + | | - | | - | | + | | - | | - | | + | | + | | *003 | |
| HOM-2 | + | | - | | - | | + | | - | | - | | + | | + | | *003 | |
| HOR | + | *00102, *048 | - | - | - | - | *002 | *002 | - | | - | | + | *003 | *002 | *002 | *003 | *01002, *015 |
| JHAF | + | *00901, *026 | - | - | - | - | *001 | *001 | - | - | - | - | + | *002, *00203 | *00302 | *00302 | *003 | *00302 |
| JVM | + | *007, *00801 | + | *001 | + | *003 | + | *001 | - | - | - | - | + | *005 | + | *00302 | *001, *003 | *001, *00302 |
| KAS011 | + | *00901, *01302 | - | - | - | - | *001, *002 | *001, *002 | - | - | - | - | + | *002, *00203 | *002, *00302 | *002, *00302 | *003 | *00302, *006 |
| KAS116 | + | *01302, *01501 | - | - | - | - | + | *001 | - | - | - | - | + | *002 | + | *00302 | *003 | *00302 |
| LBUF | + | *00301, *00901 | + | *001 | *003 | *003 | *001 | *001 | - | - | - | - | + | *00203 | *00302 | *00302 | *001, *003 | *00302, *009 |
| LUY | + | *00101, *02701 | - | - | - | - | + | *001, *005 | - | - | - | - | + | *002, *016 | + | *00302 | *003 | *00302 |
| MOU | + | *00207, *00801 | - | - | - | - | *001 | *001 | - | - | - | - | + | *002, *005 | *00302 | *00302 | *003 | *00302 |
| OLGA | + | | - | | - | | + | | - | | - | | + | | + | | *003 | |
| PE117 | + | | - | | - | | *001, *002 | | - | | - | | + | | *002, *00302 | | *003 | |
| PF04015 | + | *01402 | + | *001 | + | *003 | - | - | - | | - | | + | - | - | - | *001/ 002 | *001 |
| RSH | *0040202 , *0090101 | *00402, *0901 | *0010109 | *001 | *0010104 | *001 | *0010107 | *001 | *004 | *004 | - | | *0020105 | *002, *009 | *0030209 , *01201 | *00302, *01201 | *0030401 , *008 | *00304, *008 |
| SAVC | + | | - | | - | | *001 | | - | | - | | + | | *00302 | | *003 | |
| SPO010 | + | *00206 | - | - | - | - | + | *001 | - | - | - | - | + | *00203 | + | *00302 | *003 | *00302 |
| T7526 | 00801, 00802 | *00901 | 0010110 | - | 0030107 | - | 0010108 | *001 | - | - | 0020102 | - | 0020106 | *002 | 0030205 | *00302 | *003 | *00302 |
| VAVY | + | *00206, *017 | - | - | *011 | - | + | *001, *002 | - | - | - | - | + | *00203, *003 | + | *002, *00302 | *003 | *00302, *006 |
| WT51 | + | *00103, *036 | + | *001 | + | *001 | + | *002 | + | *002 | + | *00103/*002 | + | *004, *018 | + | *002, *00401 | + | *00301, *006 |
| WDV | + | *00301, *00901 | + | *001 | *003 | *003 | *001 | *001 | - | - | + | *002 | + | *002 | *00302 | *00302 | *001, *003 | *00302, *009 |
| YAR | + | *00102, *044 | - | - | - | - | *001, *002 | *001, *002 | - | - | - | - | + | *002, *003 | *002, *00302 | *002, *00302 | *003 | *00302, *006 |

**Supplemental Table 1: KIR genotyping of thirty reference B-EBV cell lines from the 10th International Histocompatibility Workshop (IHW). Results are presented according to the centromeric or telomeric localization of KIR genes on human genome. "+" indicates the presence of a specific KIR gene. "-" indicates the absence of a specific KIR gene. Allelic KIR typing were obtained either from IPD/KIR database[1] or from exome data[2] [47].**

| | Telomeric KIR genes | | | | | | | | | | | | | | | | | |
|---|---|---|---|---|---|---|---|---|---|---|---|---|---|---|---|---|---|---|
| | 2DL4 | | 3DL1 | | 3DS1 | | 2DL5A | | 2DS3 | | 2DS5 | | 2DS1 | | 2DS4 | | 3DL2 | |
| ID | IPD[1] | *exome* [2] | IPD[1] | *exome* [2] | IPD[1] | *exome* [2] | IPD[1] | *exome* [2] | IPD[1] | *exome* [2] | IPD[1] | *exome* [2] | IPD[1] | *exome* [2] | IPD[1] | *exome* [2] | IPD[1] | *exome* [2] |
| AMAI | *00801, 0080102 | *00801, *00801 | *00101 | *001 | - | - | - | - | - | - | - | - | - | - | *003 | *003 | *001 | *00101 |
| AMALA | *00102, *005 | *00102, *00501 | *003 | *01502 | *013 | *013 | *001 | *001 | - | | *002 | *002 | *002 | *002 | *00101 | *00101 | *002, *007 | *00201, *00701 |
| BOB | + | | + | | + | | + | | - | | + | | + | | *001/ 002 | | + | |
| BRIP | + | | + | | + | | + | | + | | - | | + | | *003-006 | | + | |
| CALOGERO | + | *00801, *00802 | + | *001, *00401 | - | - | - | - | - | - | - | - | - | - | *003-006 | *003, *006 | + | *00101, *076 |
| COX | *00501 | *00501, *011 | - | *00501 | *055 | *055 | *0010101 | *001 | - | - | *0020103 | *002 | *0020103 | *002 | - | *010 | *0070102 | *00103, *00701 |
| DEU | *00202, *005 | *00801, *011 | *00101, *005 | *001, *00501 | - | - | - | - | - | - | - | - | - | | *003 | *003, *010 | *010, *011 | *010, *011 |
| DKB | + | *00102, *00103 | + | *002, *020 | - | - | - | - | - | - | - | - | - | - | *001/ 002 | *00101 | + | *00201, *00902 |
| HO301 | + | | + | | - | | - | | + | | - | | - | | *001/ 002 | | + | |
| HID | + | | + | | - | | - | | - | | - | | - | | *001/ 002 | | + | |
| HOM-2 | *0080101 , *0080201 | | + | | - | | - | | - | | - | | - | | *003, *006 | | *010, *021 | |
| HOR | *005 | *00501 | - | | *013 | *013 | *001 | *001 | - | | *002 | *002 | *002 | *002 | - | - | *010, *021 | *00701, *021 |
| JHAF | *011 | *011 | *005 | *00501 | - | - | - | - | - | - | - | - | - | | *003 | *010 | *001, *010 | *00103, *010 |
| JVM | + | *00103, *00801 | + | *001, *008 | - | - | - | - | - | - | - | - | - | - | *003-006 | *003 | + | *00101, *00901 |
| KAS011 | *00102, *005 | *00103, *00501 | *008 | *008 | *013 | *013 | *001 | *001 | - | - | *002 | *002 | *002 | *002 | *003 | *003 | *007, *009 | *00701, *00902 |
| KAS116 | + | *011 | + | *00501 | - | - | - | - | - | - | - | - | - | - | *003, *006 | *010 | + | *00103, *010 |
| LBUF | *00102, *005 | *00102, *011 | *002, *005 | *002, *00501 | - | - | - | - | - | - | - | - | - | - | *00101, *003 | *00101, *010 | *001, *002 | *00103, *00201 |
| LUY | + | *00801, *011 | + | *00401, *00501 | - | - | - | - | - | - | - | - | - | - | *003, *006 | *006, *010 | + | *00103, *00501 |
| MOU | *0080101 | *00801 | *00101, *004 | *001, *00401 | - | - | - | - | - | - | - | - | - | - | *003, *006 | *003, *006 | *010, *011 | *01101, *01004 |
| OLGA | + | | + | | + | | + | | - | | + | | + | | *003-006 | | + | |
| PE117 | *00201, *005 | | *004 | | *013 | | *001 | | - | | *002 | | *002 | | *006 | | *005, *007 | |
| PF04015 | + | *011 | + | *00501 | - | - | - | - | - | | - | - | - | - | *003-006 | *010 | + | *00103 |
| RSH | *0010307 , *011 | *00103, *011 | *0050102 , *01701 | *00501, *017 | - | - | - | - | - | | *005 | *006 | - | - | *0010108 , *010 | *00101, *010 | *010, *023 | *010, *023 |
| SAVC | *00201, *00102 | | *003, *004 | | - | | - | | - | | - | | - | | *00101, *006 | | *002, *003 | |
| SPO010 | + | *011 | + | *00501 | - | | - | - | - | - | - | - | - | - | *003-006 | *010 | + | *00103 |
| T7526 | *0010201 , *00501 | *00103, *00501 | 0150203 | *01502 | *0130103 | *013 | + | *001 | 0020102 | - | + | *002 | *0020106 | *002 | *0010107 | *00101 | 0105, *007 | *00201, *00701 |
| VAVY | *011 | *011 | + | *00501 | - | | - | - | - | - | - | - | - | - | *003, *006 | *010 | + | *00103 |
| WT51 | + | *00501 | - | - | + | *013 | + | *001, *005 | + | *00103/*0 02 | + | *002 | + | *002 | - | - | + | *00701 |
| WDV | *00501 | *00501 | - | | *013 | *013 | *005 | *005 | + | *002 | - | - | *002 | *002 | - | - | *007 | *00701 |
| YAR | *00102,*0 05 | *00102, *011 | *002, *005 | *002, *00501 | - | - | - | - | - | - | - | - | - | - | *00101, *003 | *00101, *010 | *001, *002 | *00103, *00201 |

**Supplemental Table 1: KIR genotyping of thirty reference B-EBV cell lines from the 10th International Histocompatibility Workshop (IHW). Results are presented according to the centromeric or telomeric localization of KIR genes on human genome. "+" indicates the presence of a specific KIR gene. "-" indicates the absence of a specific KIR gene. Allelic KIR typing were obtained either from IPD/KIR database[1] or from exome data[2] [47].**
