# Supplementary material for: Killer Immunoglobulin-Like Receptor Allele Determination Using Next-Generation Sequencing Technology
Source: Front Immunol. 2017 May 19;8:547. doi: 10.3389/fimmu.2017.00547 (PMC5437120; doi:10.3389/fimmu.2017.00547)
Supplement: Supplementary file 4 [file table_2.pdf]

| KIR gene | Reference allele | Genomic length (bp) |
|---|---|---|
| KIR2DL1 | 2DL1*0020101 | 14739 |
| KIR2DL2 | 2DL2*0010101 | 14812 |
| KIR2DL3 | 2DL3*0010101 | 14761 |
| KIR2DL4 | 2DL4*0010201 | 11177 |
| KIR2DL5 | 2DL5A*0010101 | 9901 |
| KIR2DL5 | 2DL5B*0020101 | 9850 |
| KIR2DS1 | 2DS1*0020101 | 14720 |
| KIR2DS2 | 2DS2*0010101 | 14577 |
| KIR2DS3 | 2DS3*0010301 | 15103 |
| KIR2DS4 | 2DS4*0010101 | 16092 |
| KIR2DS5 | 2DS5*0020101 | 15248 |
| KIR3DL1 | 3DL1*0010101 | 14546 |
| KIR3DL2 | 3DL2*0010101 | 17009 |
| KIR3DL3 | 3DL3*0030101 | 12084 |
| KIR3DS1 | 3DS1*0130101 | 14932 |
| KIR2DP1 | 2DP1*0010201 | 13128 |
| KIR3DP1 | 3DP1*0030101 | 4236 |

**Supplemental Table 2:** KIR genomic lengths in base pairs (bp) of one reference allele for all KIR genes. KIR alleles were named according to the last nomenclature from IPD.KIR database (http://www.ebi.ac.uk/cgi-bin/ipd/kir/).
