# Supplementary material for: Killer Immunoglobulin-Like Receptor Allele Determination Using Next-Generation Sequencing Technology
Source: Front Immunol. 2017 May 19;8:547. doi: 10.3389/fimmu.2017.00547 (PMC5437120; doi:10.3389/fimmu.2017.00547)
Supplement: Supplementary file 5 [file table_3.pdf]

	Mean of coverage	% Mapping
KIR3DL3	198.2	95.0
KIR2DS2	182.8	95.5
KIR2DL2	156.0	92.9
KIR2DL3	96.0	90.9
KIR2DL5B	560.1	94.1
KIR2DS3	84.2	89.2
KIR2DP1	201.3	98.2
KIR2DL1	132.2	96.0
KIR3DP1	2373.3	98.2
KIR2DL4	605.8	97.9
KIR3DL1	107.7	89.0
KIR3DS1	71.1	95.5
KIR2DL5A	undetermined	undetermined
KIR2DS5	72.5	92.9
KIR2DS1	97.5	95.8
KIR2DS4	62.5	86.6
KIR3DL2	63.6	86.2
**All loci**	**316.55**	**93.37**

Supplemental Table 3: Mean coverage and percentage of mapping obtained for each KIR gene using Profiler software
